# What Do We Have to Know about PD-L1 Expression in Prostate Cancer? A Systematic Literature Review. Part 3: PD-L1, Intracellular Signaling Pathways and Tumor Microenvironment

**DOI:** 10.3390/ijms222212330

**Published:** 2021-11-15

**Authors:** Andrea Palicelli, Stefania Croci, Alessandra Bisagni, Eleonora Zanetti, Dario De Biase, Beatrice Melli, Francesca Sanguedolce, Moira Ragazzi, Magda Zanelli, Alcides Chaux, Sofia Cañete-Portillo, Maria Paola Bonasoni, Alessandra Soriano, Stefano Ascani, Maurizio Zizzo, Carolina Castro Ruiz, Antonio De Leo, Guido Giordano, Matteo Landriscina, Giuseppe Carrieri, Luigi Cormio, Daniel M. Berney, Jatin Gandhi, Valerio Copelli, Giuditta Bernardelli, Giacomo Santandrea, Martina Bonacini

**Affiliations:** 1 Pathology Unit, Azienda USL-IRCCS di Reggio Emilia, 42123 Reggio Emilia, Italy; Alessandra.Bisagni@ausl.re.it (A.B.); Eleonora.Zanetti@ausl.re.it (E.Z.); Moira.Ragazzi@ausl.re.it (M.R.); Magda.Zanelli@ausl.re.it (M.Z.); mariapaola.bonasoni@ausl.re.it (M.P.B.); valerio.copelli@ausl.re.it (V.C.); giuditta.bernardelli@ausl.re.it (G.B.); giacomo.santandrea@ausl.re.it (G.S.); 2 Clinical Immunology, Allergy and Advanced Biotechnologies Unit, Azienda USL-IRCCS di Reggio Emilia, 42123 Reggio Emilia, Italy; Stefania.Croci@ausl.re.it (S.C.); Martina.Bonacini@ausl.re.it (M.B.); 3 Department of Pharmacy and Biotechnology (FABIT), University of Bologna, 40126 Bologna, Italy; dario.debiase@unibo.it; 4 Fertility Centre, Department of Obstetrics and Gynecology, Azienda USL-IRCCS di Reggio Emilia, 42123 Reggio Emilia, Italy; Beatrice.Melli@ausl.re.it; 5 Clinical and Experimental Medicine PhD Program, University of Modena and Reggio Emilia, 41121 Modena, Italy; Carolina.CastroRuiz@ausl.re.it; 6 Pathology Unit, Policlinico Riuniti, University of Foggia, 71122 Foggia, Italy; francesca.sanguedolce@unifg.it; 7 Department of Scientific Research, School of Postgraduate Studies, Norte University, Asunción 1614, Paraguay; alcideschaux@uninorte.edu.py; 8 Department of Pathology, University of Alabama at Birmingham, Birmingham, AL 35294, USA; scaneteportillo@uabmc.edu; 9 Department of Pathology, Case Western Reserve University, Cleveland, OH 44106, USA; alessandra.soriano@ausl.re.it; 10 Gastroenterology Division, Azienda USL-IRCCS di Reggio Emilia, 42123 Reggio Emilia, Italy; 11 Pathology Unit, Azienda Ospedaliera Santa Maria di Terni, University of Perugia, 05100 Terni, Italy; s.ascani@aospterni.it; 12 Haematopathology Unit, CREO, Azienda Ospedaliera di Perugia, University of Perugia, 06129 Perugia, Italy; 13 Surgical Oncology Unit, Azienda USL-IRCCS di Reggio Emilia, 42123 Reggio Emilia, Italy; Maurizio.Zizzo@ausl.re.it; 14 Molecular Diagnostic Unit, Azienda USL Bologna, Department of Experimental, Diagnostic and Specialty Medicine, University of Bologna, 40138 Bologna, Italy; antonio.deleo@unibo.it; 15 Medical Oncology Unit, Department of Medical and Surgical Sciences, University of Foggia, 71122 Foggia, Italy; guido.giordano@unifg.it (G.G.); matteo.landriscina@unifg.it (M.L.); 16 Department of Urology and Renal Transplantation, University of Foggia, 71122 Foggia, Italy; giuseppe.carrieri@unifg.it (G.C.); luigi.cormio@unifg.it (L.C.); 17 Barts Cancer Institute, Queen Mary University of London, London EC1M 5PZ, UK; daniel.berney@nhs.net; 18 Department of Pathology and Laboratory Medicine, University of Washington, Seattle, WA 98195, USA; jgandhi@uw.edu

**Keywords:** PD-L1, prostate, cancer, signaling pathways, tumor microenvironment, target-therapy, immunotherapy, checkpoint inhibitors

## Abstract

The tumor microenvironment (TME) includes immune (T, B, NK, dendritic), stromal, mesenchymal, endothelial, adipocytic cells, extracellular matrix, and cytokines/chemokines/soluble factors regulating various intracellular signaling pathways (ISP) in tumor cells. TME influences the survival/progression of prostate cancer (PC), enabling tumor cell immune-evasion also through the activation of the PD-1/PD-L1 axis. We have performed a systematic literature review according to the PRISMA guidelines, to investigate how the PD-1/PD-L1 pathway is influenced by TME and ISPs. Tumor immune-escape mechanisms include suppression/exhaustion of tumor infiltrating cytotoxic T lymphocytes, inhibition of tumor suppressive NK cells, increase in immune-suppressive immune cells (regulatory T, M2 macrophagic, myeloid-derived suppressor, dendritic, stromal, and adipocytic cells). IFN-γ (the most investigated factor), TGF-β, TNF-α, IL-6, IL-17, IL-15, IL-27, complement factor C5a, and other soluble molecules secreted by TME components (and sometimes increased in patients’ serum), as well as and hypoxia, influenced the regulation of PD-L1. Experimental studies using human and mouse PC cell lines (derived from either androgen-sensitive or androgen-resistant tumors) revealed that the intracellular ERK/MEK, Akt-mTOR, NF-kB, WNT and JAK/STAT pathways were involved in PD-L1 upregulation in PC. Blocking the PD-1/PD-L1 signaling by using immunotherapy drugs can prevent tumor immune-escape, increasing the anti-tumor activity of immune cells.

## 1. Introduction

The tumor microenvironment (TME) includes immune cells, stromal/mesenchymal cells (such as activated fibroblasts or adipocytes), blood vessels, extracellular matrix, as well as cytokines, chemokines, and other soluble factors released by each TME-component. The interactions between these different TME elements influence tumor growth and cancer survival/progression, enabling tumor cell immune-evasion. TME influences the immunogenicity of various tumor types, including prostate cancer (PC) [1,2,3].

Programmed death-1 (PD-1) is a type I transmembrane glycoprotein of the CD28/CTLA-4 family, encoded by *PDCD1* gene (located on chromosome 2). Its expression is inducible upon cell activation, and strictly related to hematopoietic cells (activated T, B, NK cells and monocytes) [2,3]. The PD-1 protein consists of an extracellular IgV-type domain, a transmembrane region and an intracellular tail characterized by immunoreceptor tyrosine-based inhibitory and switch motifs responsible for the intracellular signaling cascade [1,2,3].

In humans, two PD-1 ligands have been identified: PD-L1 and PD-L2 [2,3]. They are type I transmembrane glycoprotein B7 family members transcribed by *CD274* and *PDCD1LG2* genes, respectively: both genes are located on chromosome 9. Their expression in hematopoietic and non-hematopoietic cells is inducible by microenvironmental conditions; PD-L1 shows a higher and more widespread expression profile than PD-L2, which is more frequently expressed on antigen-presenting cells (dendritic cells and macrophages) [2,3]. PD-L1 and PD-L2 proteins consist of IgC and IgV-type extracellular domains, a transmembrane region and an intracellular tail without any canonical signaling motifs [2,3].

The PD-1/PD-L1 signaling pathway is involved in the regulation of the tumor immune escape by its effects on inflammatory cells (T and B lymphocytes, NK cells, macrophages, dendritic cells) and other TME components. It inhibits the tumor-infiltrating lymphocytes (TILs) and NK cells function/activation, favoring TILs apoptosis, and influencing the T helper and myeloid cell differentiation; moreover, the secretion of immunosuppressive cytokines is promoted, decreasing the production of effector cytokines [1,2,3,4]. The identification of novel prognostic markers strictly linked to the development of targeted therapies is urgently required in various tumors, including PC [4,5,6,7]. Blocking PD-L1 using checkpoint inhibitors may restore the anti-tumor activity of immune cells [2,3,8,9].

To delineate the intracellular signaling pathways and the extracellular TME factors involved in PD-L1 expression, we have performed a systematic literature review of human tissue-based studies (immunohistochemical, molecular, etc.), experimental research (cell lines, mouse models), and closed clinical trials.

## 2. Results

### 2.1. Literature Review Results

**Figure 1:**
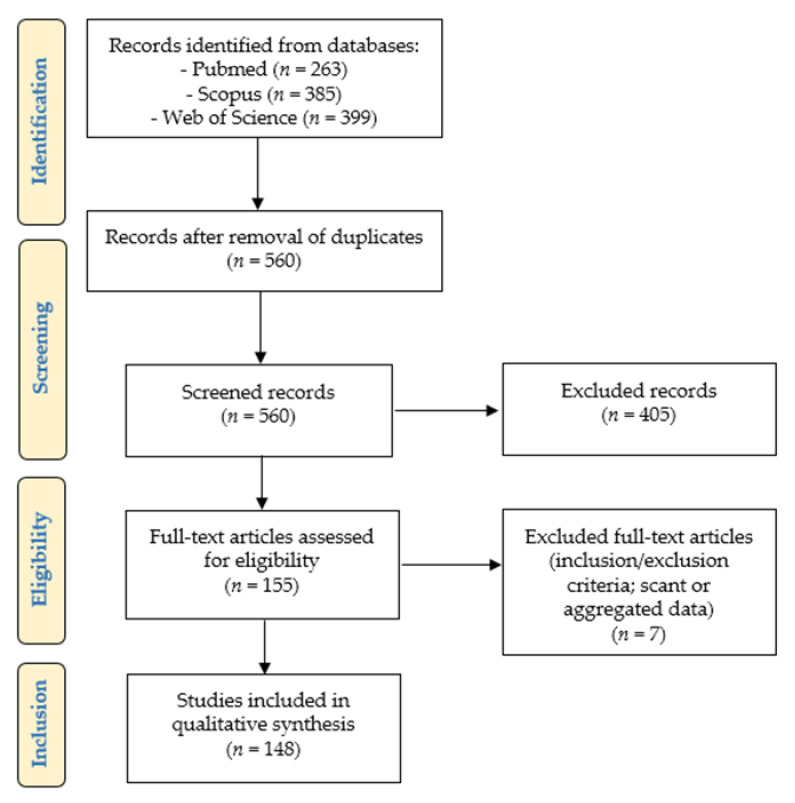
Systematic review of the literature: PRISMA flow-chart.

Figure 1 presents the “Preferred Reporting Items for Systematic Reviews and Meta-Analyses” (PRISMA) (http://www.prisma-statement.org/ accessed on 8 May 2021) flow chart, reporting a summary of the method and results of our systematic literature review.

We identified 263 articles on Pubmed (https://pubmed.ncbi.nlm.nih.gov; accessed on 8 May 2021), 385 articles on Scopus (https://www.scopus.com/home.uri; accessed on 8 May 2021), and 399 articles on Web of Science databases (https://login.webofknowledge.com; accessed on 8 May 2021). After duplicates removal, 560 records underwent first-step screening of titles and abstracts: 155 articles were found to be eligible for our study, and they were retrieved in full text format. After reading them, 7/155 papers were excluded, as they did not satisfy the inclusion criteria, or because they presented scant or aggregated data. 148 articles were finally included in our study [8,9,10,11,12,13,14,15,16,17,18,19,20,21,22,23,24,25,26,27,28,29,30,31,32,33,34,35,36,37,38,39,40,41,42,43,44,45,46,47,48,49,50,51,52,53,54,55,56,57,58,59,60,61,62,63,64,65,66,67,68,69,70,71,72,73,74,75,76,77,78,79,80,81,82,83,84,85,86,87,88,89,90,91,92,93,94,95,96,97,98,99,100,101,102,103,104,105,106,107,108,109,110,111,112,113,114,115,116,117,118,119,120,121,122,123,124,125,126,127,128,129,130,131,132,133,134,135,136,137,138,139,140,141,142,143,144,145,146,147,148,149,150,151,152,153,154,155].

### 2.2. Experimental Studies: Intracellular Signaling Pathways Involved in PD-L1 Expression in PC

**Figure 2:**
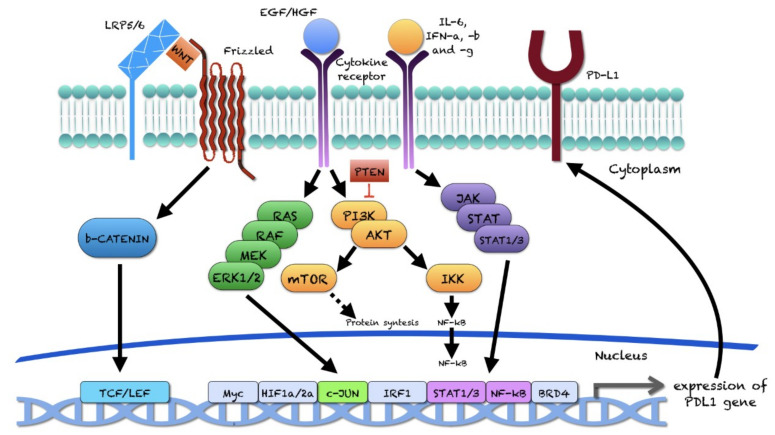
Intracellular signaling pathways involved in PD-L1 expression.

Many articles investigated the role of the PD-1/PD-L1 axis on tumor and immune cells in PC cell lines and mouse models. Experimental studies revealed that the intracellular ERK/MEK, Akt-mTOR, NF-kB, WNT, and JAK/STAT pathways were involved in PD-L1 regulation in PC (Figure 2) [110].

The different signaling pathways were investigated by variably using human and/or mouse PC cell lines, derived from either androgen-sensitive or androgen-resistant PCs. In particular, the ERK/MEK and Akt-mTOR pathways were studied only in human metastatic androgen-independent cell lines (PC3, DU145) [105,121], while the NF-kB and WNT pathways were additionally investigated in human metastatic androgen-sensitive cell lines (LNCaP) [53,117,145]. Finally, the JAK/STAT pathway was explored not only in human metastatic androgen-sensitive (LNCaP) and androgen-independent cell lines (PC3, DU145, C4-2, CWR22RV1, LASCPC, NCI-H660), but also in a primary mouse cell line (TRAMP-C2) [13,65,97,126,131,134,136,143]. All these studies agreed that the activation of ERK, NF-kB and JAK/STAT pathways leads to PD-L1 upregulation, regardless of the tumor sample type (primary vs. metastatic) and of the androgen responsiveness/resistance of the analyzed PC-cell lines. Indeed, treatment of PC-cell lines with NF-kB, MEK, JAK, or STAT inhibitors down-modulated PD-L1-expression. It has been documented that PTEN upregulation caused inhibition of mTOR and PD-L1 in mice injected with PC-cells overexpressing chemerin (a chemoattractant protein and PTEN-activator). The AKT-mTOR inhibition and the chemerin-induced PD-L1 downregulation caused a significant reduction of tumor growth in these preclinical models [105].

### 2.3. Data from The Cancer Genome Atlas (TCGA) Analysis

**Figure 3:**
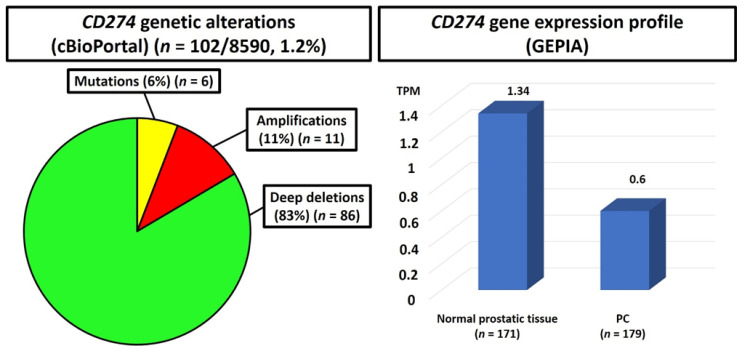
Results of big data analysis: *CD274* genetic alterations ((**left**): cBioPortal; https://www.cbioportal.org/ (accessed on 12 August 2021)) and gene expression profile ((**right**): GEPIA database; http://gepia.cancer-pku.cn/index.html (accessed on 12 August 2021)). PC: prostate cancer; TPM: transcripts per millionset. On the right, the median TPM values of *CD274* gene expression in normal prostatic tissue and PC are repoted above the respective histograms.

Some articles investigated the role of PD-L1 in human patients using data retrieved from the TCGA. The *CD274* gene, encoding for PD-L1, is located on chromosome 9p24.1: genomic rearrangements may upregulate *CD274* expression, leading to enhanced immune escape of tumor cells [156]. Analysis of the data available on cBioPortal online database (https://www.cbioportal.org/; accessed on 1 October 2021) revealed that 102/8590 (1.2%) of PC cases included in this dataset harbored genetic alterations of *CD274* gene, including mutations (6%), amplifications (11%) and deep deletions (83%) (Figure 3).

Analysis of GEPIA database (http://gepia.cancer-pku.cn/index.html; accessed on 1 October 2021) revealed that the median values of *CD274* gene expression in normal prostatic tissue was higher than in PC (1.34 vs. 0.6 transcripts per millionset) (Figure 3). Gevensleben et al. [86] analyzed the methylation of PD-L1 promoter (mPD-L1) in a TCGA-training cohort of 498 PCs and a validation group of 299 radical prostatectomy specimens: normal prostatic tissues showed lower levels of mPD-L1 compared to PC-samples. In another study, PD-L1 mRNA expression was significantly lower in PCs (*n* = 492, TCGA set; *n* = 110, radical prostatectomy and biopsy samples of the authors’ series) than in bladder (*n* = 404, TCGA set) or renal carcinomas (*n* = 534, TCGA set): only the abstract was available [85].

Chromosome 9p gains are common DNA copy number aberrations, significantly associated with advanced tumor stage and regional lymph node metastases in sporadic PCs [157,158]. Budczies et al. identified a 7.8-Mbp region of 38 genes located in chromosome 9p24, including genes regulating cell cycle and immune-response in PC (such as *PD-L1*, *PD-L2* and *JAK2*). Across 22 tumor types originating from various districts (genito-urinary, gastro-intestinal, gynecological, head and neck, brain, endocrine, pulmonary, etc.) (TCGA data), this region was co-amplified with PD-L1 in >80% of tumors with focal *PD-L1* copy number gains (CNGs). The authors found that PCs showed significant PD-L1 mRNA expression changes (1.8-fold change, FC, *p* = 0.0009), PD-L2 (1.6 FC, *p* = 0.002), and JAK2 (1.4 FC, *p* = 0.00017) in cases with *PD-L1* CNGs, compared to tumors lacking these gains [68]. PCs also showed upregulation of immune-system related genes (including *ADAMDEC1*, *APLN*, *CCL4*, *CCL8, CXCL10, CXCL11, FCGR3A, GBP5, IDO1, IFI44L, IFNG, JAK2, KIF2C, MELK, PD-L1, PD-L2*) and of genes regulating the cellular proliferation (including *ASPM, CDC20, CDCA8, CDKN2A, CENPA, CENPF, CENPI, CLSPN, FAM83D, GTSE1, HAUS6, IFNG, KIF18B, KIF2C, MELK, MYBL2, NUF2*, *PLK1, SPC24, TPX2, TTK, UHRF2*): however, it was unclear which specific genes were over-expressed in each of the 2 categories [68].

Meng et al. [19] analyzed various cohorts of PCs (also derived from TCGA) for a total number of 1557 cases, which were divided into 3 classes (immune-activated, immune-suppressed, and non-immune). The signatures of WNT/TGF-β, TGF-β 1, and extracellular matrix cytokines (C-ECM) were more enriched in the immune-suppressed (stromal-activated) subtype than in the non-immune class (*p* < 0.05): these cases showed increased expression of IL-11, TGFB1, and TGFB2, and high expression of tumor-infiltrating regulatory T cell (Treg) signatures (*p* < 0.05). The immune-activated class revealed high enrichment of Th17 cell infiltrate signature, the best recurrence-free survival outcomes, and potential benefit from anti-PD-1/PD-L1 treatment. Copy number alterations of immune checkpoints (such as PD-1, PD-L1, LGALS9, and CD48) positively correlated to immunocytes infiltrate. The immune class showed higher density of TILs (*p* = 0.001) and CD163+ cells (immune-suppressed group, *p* < 0.0001; immune-activated subtype, *p* = 0.0018), and increased PD-L1 expression (*p* < 0.001). In 2013, the U.S. FDA (Food and Drug Administration) approved the PAM50 molecular classifier for the clinical prognostic subclassification of breast cancer patients into luminal A, luminal B, HER2-enriched, and basal-like categories [159]. Meng et al. [19] applied this classifier to PC patients, finding that the immune-suppressed subtype included more luminal B-like cases, while the immune-activated class comprised more luminal A-like PCs: the second group showed higher expression of immune checkpoint (PD-L1 and CTLA-4) and chemokine genes (*CXCL9* and *CXCL10*).

CTHRC1 (Collagen Triple Helix Repeat Containing 1) encodes a protein potentially involved in vascular remodeling: CTHRC1 mRNA expression positively correlated to increased PD-1 and PD-L1 levels in a TCGA cohort [120].

TCGA-studies concerning the epigenetic regulation of PD-L1 in PC are better presented and discussed in other parts of our review (see Materials and Methods): here we report a brief summary of relevant data. In a series (*n* = 35), PD-L1 RNA levels were higher in metastatic (vs. primary) PC-samples, correlating to *MLL3* expression (a histone modifier): this association was confirmed also by TCGA data (*p* < 0.01) [56]. Analysis of TCGA datasets found that PD-L1 expression was positively associated with LIF levels, which were stimulated by *RNF165* (RING finger protein 165) gene transcripts, including a novel long non-coding RNA (lncAMPC) [104]. PCs overexpressing WDR5 (histone methylation regulator) were more frequently PD-L1+ by immunohistochemistry in a series of 262 PCs: a positive correlation between WDR5 and PD-L1 mRNA levels was also confirmed by a TCGA set (*n* = 374) [10]. Expression of PD-L1 and its positive regulators (*EP300*, *CREBBP*, *IRF-1*, and *BRD4*) negatively correlated with Grade Group in another study, including TCGA data [112]. Conversely, negative PD-L1 regulators (HDAC1, HDAC2, and HDAC3) seemed insignificant during cancer progression. Moreover, an inverse association was found between: (1) *PD-L1*, *EP300*, and *CREBBP* expression and overall survival; (2) PD-L1 and PD-1 expression and tumor purity; (3) PD-L1 and PD-1 expression and increased tumor-infiltrating immune cells [112]. In the abovementioned study of Gevensleben et al. [86], high mPD-L1 (*p* = 0.008) and high PD-L1 protein expression (*p* = 0.002) (analyzed as continuous variables) both correlated with shorter biochemical recurrence-free survival. mPD-L1 was also significantly associated with pT stage (*p* = 0.010) and Grade Group (*p* = 0.001). miR-197 and miR-200a-c positively correlated to PD-L1 mRNA levels, being inversely associated with mPD-L1. miR-34a inversely correlated to mPD-L1 and mRNA expression.

### 2.4. Experimental Studies: Overview of Extracellular Factors Involved in PD-L1 Regulation in PC

The extracellular regulators of PD-L1 expression, investigated in the published studies, included cytokines/chemokines (IL-6, Il-27, IL-17, IFN-γ, TNF-α, chemerin), complement factors (C5a), soluble factors released by stromal cells (AREG, IL-6) or adipocytes, and hypoxia. Table 1 summarizes the effects of PD-L1 extracellular regulators in PC cell lines and experimental studies.

Except for chemerin, all the abovementioned factors induced PD-L1 activation/upregulation. Soluble PD-1 caused an increase in docetaxel resistance of PC-cell lines in a study [149]. AREG favored tumor cell proliferation, migration and invasion [121]. Soluble factors released from adipocytes or macrophages, and hypoxic stress, decreased the NK cell cytotoxicity [65,134,136]. Chemerin favored an increase of the T cell toxicity [105]. The number of performed studies widely varied among the abovementioned investigated factors. Moreover, some PC cell lines were preferentially used in experimental studies, while other cells lines were rarely tested.

Concerning cytokines, the effects of IFN-γ were the most investigated in both human and mouse cell lines. Increased serum levels of various cytokines were also observed in the circulation of castration-resistant PCs (CRPCs) patients, including FGF, EGF, IL-6, IL-10, GM-CSF, IGF, and TGF-β, sometimes along with their respective receptors: they have a role in promoting tumor progression and/or immune evasion [44,160]. IFN-γ, IL-5, IL-10, MIP-1α, and TNF-α were also increased in treatment-sensitive patients [44]. To our review, IL-6 [135], IL-17 [145], TNF-α [145], IL-27 [151] upregulated PD-L1 in cell lines.

### 2.5. IFN-γ

IFN-γ is released by CD8+ T cells, NK cells and macrophages to enhance their effector functions by stimulating antigen processing, and increasing TILs differentiation [161]. However, IFN-γ can be captured and used by tumor cells to paralyze T cells. To our review, concerning cytokines, the effects of IFN-γ were the most investigated in both human and mouse PC cell lines.

The JAK-STAT signaling pathway is frequently over-activated in PC cell lines (especially if metastatic and androgen-resistant). PC cell lines can be classified as sensitive or insensitive to IFN-γ. Indeed, most of the experiments about IFN-γ treatment were performed in metastatic androgen-insensitive PC cell lines. The published studies reported that treating IFN-γ-sensitive PC-cell lines with IFN-γ induced PD-L1 expression via upregulation of the JAK/STAT pathway. In turn, PD-L1 can suppress the proliferation and functions of NK cells and cytotoxic T lymphocytes, causing T cell apoptosis [108,112]. These data also explained (at least in part) why a response to IFN-γ was observed in the majority of in vitro experiments.

On the other hand, literature data also reported IFN-γ insensitive PC cell lines. For some of them, alterations in the JAK/STAT pathway (deletions, inactivations, etc.) were described, including the loss of *JAK1* gene expression in LNCaP cell lines [162]. According to our review, we were not able to find significant data correlating mutations in JAK/STAT pathway and PD-L1 in IFN-γ-insensitive PC cell lines. Table 2 reports the details of the performed studies, including IFN-γ concentrations, time of treatment and the techniques used for IFN-γ detection.

In the experimental study of Zhang et al., high RelB (a driver of the NF-κB alternative pathway) and PD-L1 levels correlated to the Gleason score of PCs. IFN-γ may induce PD-L1 expression by promoting NF-κB pathway activation and RelB nuclear translocation, resulting in PD-L1 transcriptional activation [137].

Major histocompatibility complex (MHC) class I molecules promote the presentation of non-self antigens on cancer cell surface, favoring their immune recognition by CD8+ cytotoxic T lymphocytes: PC cells frequently downregulate MHC class I molecules to evade the immune detection [143,163,164,165,166,167,168]. Both HLA-ABC and PD-L1 are upregulated by IFN-γ via STAT1 phosphorylation [168]. In the study of Liu et al., a JAK2-inhibitor failed to induce HLA-ABC and PD-L1 expression, while treatment with the MEK (mitogen-activated protein kinase/extracellular signal-regulated kinase) inhibitor PD0325901 did not upregulate HLA-ABC and PD-L1 [143].

Analyzing acinar and small cell neuroendocrine PCs (SCNPCs), Sun et al. found that SCNPCs revealed the highest levels of PD-L1, IDO-1 (indoleamine 2,3-dioxygenase), and N-cadherin; globally, the first 2 markers were highly expressed in N-cadherin + PCs [13]. N-cadherin modulated the IFN-γ-Receptor/JAK1/STAT1 pathway, upregulating the IFN-γ-induced expression of JAK1, PD-L1 and IDO. It also increased the production of free fatty acids, promoting Tregs generation. Conversely, the JAK2-STAT3 signaling was only linked to IDO-1 expression in this study.

IDO catalyzes the rate-limiting step of Tryptophan catabolism to Kynurenine (an endogenous ligand of the aryl hydrocarbon receptor), regulating the acquired local and peripheral immune tolerance in physiological and pathological conditions [49,151].

### 2.6. TGF-β

Transforming growth factor β (TGF-β) is a putative mediator of castration-resistance in preclinical PC-models. TGF-β inhibits cell proliferation and induces apoptosis in prostatic epithelial cells. However, PC-cells do not express TGF-β1 receptors, and this is compounded by the overexpression of TGF-β [169]. Loss of TGF-β1 effect on PC-cells is apparently associated with more advanced tumors and greater metastatic potential. Meng et al. [19] dichotomized their series into immune-activated and immune-suppressed subtypes based on the stromal signature, represented by the activation of WNT/TGF-β, TGF-β1, and C-ECMs. Patients in the immune-suppressed group showed the worst recurrence-free survival, increased expression of IL-11, TGFB1, and TGFB2, and enrichment of tumor-infiltrating Treg signatures (*p* < 0.01), while immune-activated PCs (14.9–24.3%) might benefit from anti-PD-1/PD-L1 therapy, as to their higher expression of immune checkpoint (PD-L1 and CTLA-4) and chemokine genes (*CXCL9* and *CXCL10*).

### 2.7. IL-17 and TNF-α

IL-17 induces the intratumor infiltration by PD-1+ immune cells, and promotes PD-L1 expression by tumor cells [150]. Analyzing the PCs of *PTEN*-null mice, Yang et al. found that IL-17rc wild-type mice showed higher levels of PD-1, PD-L1, and PD-L2, developing more aggressive tumors than IL-17rc knockout mice [150].

IL-17- and TNF-α-secreting Th17 cells seemed enriched in PC, potentially favoring an immunosuppressive TME. Some authors [145,170] found that these 2 molecules induced PD-L1 protein expression in some PC cell lines (LNCaP), but only TNF-α increased the PD-L1 mRNA levels. Conversely, PD-L2 protein and mRNA expression were not upregulated. The authors favored an individual (rather than cooperative) activity of IL-17 and TNF-α via the AKT and NF-kB pathways. Indeed, NF-kB or AKT inhibitors downregulated the IL-17/TNF α–induced protein expression of PD-L1.

### 2.8. IL-27

IL-27 (IL-12 family member) is a heterodimer of p28 (IL-27A) and EBI3 (EBV-induced gene 3) chains, favoring the CD4+ T cells proliferation, and upregulating the expression of IL-12-receptor (thus inducing Th1 polarization). IL-27 directly inhibits cancer growth or invasiveness, showing immune-enhancing activity in different in vivo tumor models [151]. However, the IL-27-induced priming may convert naïve T cells into PD-L1+ Tregs, limiting the IL-17 production [171]. Carbotti et al. found that IL-27 induced low IDO (mRNA and protein) levels and PD-L1 expression in human PC cell lines (PC3).

### 2.9. IL-15

IL-15 is a pivotal cytokine in the development and homeostasis of intraepithelial and CD8+ T lymphocytes, NK, and NK/T cells: unlike IL-2, IL-15 does not expand Tregs.

In murine PC-models, the IL-15-mediated antitumor immune response seemed to recruit NK, CD8+, and CD4+ T cells in TME. Treatment of transgenic (TRAMP)-C2 murine PC models with a triple therapy (IL-15, anti-CTLA-4 and anti-PD-L1 antibodies) enhanced the CD8 + T cell cytotoxic activity and the antigen-specific IFN-γ release, inhibiting the suppressive functions of CD4+/CD25+ and CD8+/CD122+ Tregs [153]. This therapeutic combination decreased the tumor growth and prolonged the survival of tumor-bearing animals. However, response to IL-15 often resulted in overproduction of proinflammatory cytokines (such as IL-6, IL-10, TNF-α, and IFN-γ), which can sometimes lead to serious side effects in patients.

IL-10 is a key immunoregulator, suppressing T cell functions and inducing Tregs development, thus favoring tumor immune evasion and the lower frequency of T cells observed in pre-treatment patients. Combining IL-15 with anti-IL-10 or anti-TGF-β drugs failed to improve the favorable effects of IL-15. Administration of IL-15 in combination with blockade of CTLA-4 and/or PD-1 inhibited the IL-10 secretion by T-cells. Although IL-15 expanded the numbers of NK1.1+ cells, anti-asialo-GM1 (injected to deplete these cells) did not influence the tumor growth, while CD8 depletion abrogated the antitumor effects of IL-15 and triple combination treatments. Moreover, TRAMP-C2 cells express high levels of MHC class I molecules, probably limiting the NK tumor-killing efficacy [153,172].

### 2.10. IL-6

IL-6 is a proinflammatory cytokine involved in immunoregulation, cell growth and differentiation, as well as in obesity-related insulin-resistance and chronic inflammatory condition: plasma IL-6 levels positively correlated to weight gain in humans [136]. In some studies, high levels of IL-6 and IL-6 receptor (IL6R) have been associated with more aggressive PCs (disease progression, castration-resistance) [45,173].

IL-6 promotes immunosuppression and an androgen-independent phenotype [45,173]. In PC models, IL-6 activated the JAK/STAT3, MAPK (mitogen-activated protein kinase) and PI3K (phosphatidylinositol 3-kinase) pathways, as well as the androgen receptor (AR)-mediated trascription (even in the absence of androgen ligands). Moreover, IL-6 is involved in the regulation of vascular endothelial growth factor (VEGF), neuroendocrine differentiation, epithelial-mesenchymal transition and metastatic progression of PC. IL-6 induced resistance of CRPC cells to the NK cell-mediated cytotoxicity via activation of the JAK/STAT3 pathway, modulating the PD-L1 and NKG2D-ligand levels in CRPC cells [135,136,174]. Finally, IL-6 induces the development of immunosuppressive myeloid-derived suppressor cells (MDSCs) or tumor-associated macrophages (TAMs): their immunosuppressive functions occur through a STAT3-mediated mechanism [45].

### 2.11. Complement System and Hypoxia

The complement system (CS) is activated in various malignancies. C5a is a CS-product, a leukocyte chemoattractant and an inflammatory mediator binding to the C5a receptor (C5aR; CD88), which is aberrantly expressed by cancer cells. C5a is also directly released from tumor cells through a cell membrane protease. C5a-C5aR interactions enhance tumor growth, invasiveness, immune-evasion and metastatic behavior through motility activation, increased release of matrix metalloproteases, and activation of the PI3K/AKT pathway, leading to PD-L1 expression in PC-cells. C5a also recruits the MDSCs, which inhibit the antitumor CD8+ T-cell response and can express PD-L1 [18].

Hypoxia can induce neoangiogenesis, and promotes the epithelial-mesenchymal transition, invasion, and metastatic progression of PCs, decreasing the sensitivity to radiotherapy and chemotherapy [65]. Hypoxia is involved in the immune evasion mechanisms of a variety of tumors, by increasing Nanog and TGF-β1 expression, regulating miRNA levels, and reducing the immune anti-tumor activity of T cells and macrophages. In PC cells lines, hypoxic conditions favored PD-L1 upregulation and NKG2D ligands downregulation on the surface of the tumor cells, thus inhibiting the tumor killing by activated NK cells [65].

### 2.12. AREG

Cytotoxic agents frequently trigger irreparable damage in peritumoral stromal cells, generating many cells displaying a “senescence-associated secretory phenotype” (SASP). SASP cells can favor tissue homeostasis by supporting tissue repair, wound healing, and immunosurveillance, but also contribute in developing aging-related complications (atherosclerosis, osteoarthritis, physical frailty, and systemic inflammation), and play an important role in the TME [175]. SASP cells secrete various soluble factors (cytokines, chemokines, growth factors, and proteases) favoring chemoresistance and sometimes immunosuppression in the treatment-damaged TME [121]. Amphiregulin (AREG) is a ligand and activator of the epidermal growth factor receptor, released by treatment-damaged senescent stromal cells as a soluble factor through extracellular vesicles (such as exosomes). AREG potently enhances the malignant behavior of various primary and metastatic tumors [176]. Mast cell-derived AREG potentiates the immunosuppressive competency of Tregs [177].

AREG induced PD-L1 expression in PC cell lines, causing exhaustion of cytotoxic T and NK cells [121]. In PC mouse models, AREG was produced by the tumor stroma after damage (chemotherapy, ionizing radiation, etc.), conferring resistance to immunosurveillance by increasing PD-L1 expression on cancer cells [121]. AREG is a hallmark SASP molecule and potential biomarker, being detectable in the blood of post-treatment cancer patients.

## 3. Discussion

### 3.1. Tumor Microenvironment and Mechanisms of Tumor Immune-Escape Mediated by the PD-1/PD-L1 Axis: An Overview

The TME is the tumor milieu, including immune cells, stromal/mesenchymal cells (such as activated fibroblasts, adipocytes, etc.), blood vessels, and extracellular matrix, as well as cytokines, chemokines, and other soluble factors released by each TME component. The interactions between these different TME elements are necessary for cancer growth and progression, enabling tumor cell immune evasion. Studies on TME have demonstrated that cancer cells can upregulate PD-L1 expression and interact with other TME components via PD1/PD-L1 and other pathways [157].

**Figure 4:**
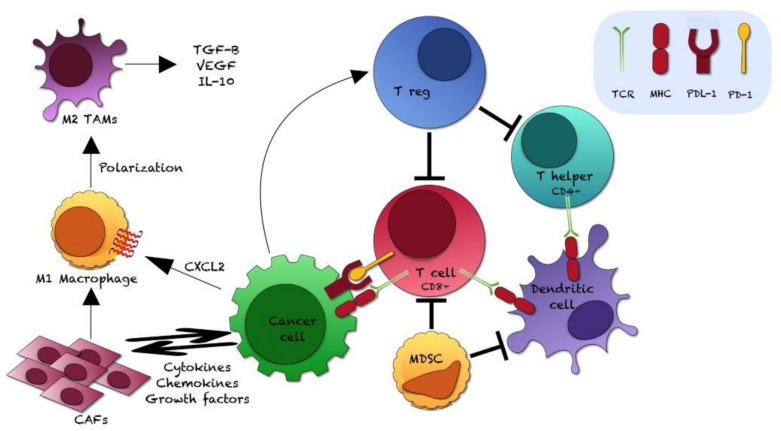
Interaction of prostate cancer cells with other cell types of the tumor microenvironment. CAFs: cancer-asssociated fibroblasts; CXCL2: Chemokine (C-X-C motif) ligand 2; IL-10: Interleukin-10; MDSC: myeloid-derived suppressor cell; MHC: major histocompatibility complex; TAMs: tumor-associated macrophages; T cell CD8+: CD8+ Cytotoxic T lymphocyte; TCR: T cell receptor; TGF-β: Tumor Growth Factor β; T helper CD4+: CD4+ helper T lymphocyte; T reg: regulatory T cell; VEGF: Vascular-Endothelial Growth Factor; PD-1: programmed death 1; PDL-1: programmed death ligand 1.

Figure 4 illustrates the interaction of tumor cells with the various cell types of the TME.

Mechanisms of immune-escape by tumor cells mediated by the PD-1/PD-L1 axis include [65,79]:

(1) Suppression or inadequate activation/boosting of TILs activity:
  - reduction of the proliferation of intratumoral cytotoxic T cells
  - induction of CD8+ T cell exhaustion: CD8+ T cells may be present but incapable to mediate cytotoxic activity
  - increase of CD8+ T cell apoptosis
  - inhibition of Tregs apoptosis.
  - B cells regulation
(2) Inhibition of function and activation of NK cells (which can kill tumor cells directly without dependence on antibodies or complement factors)
(3) Regulation of the secretion of soluble factors (inhibition of the production of effector cytokines; promotion of the secretion of immunosuppressive cytokines; chemotactic factors recruiting immunosuppressive cells; etc.)
(4) Immunosuppressive effects mediated by TAMs, MDSCs, dendritic cells and other cell-types (stromal cells, adipocytes, etc.): these cells may express PD-L1, recruit other immunosuppressive cells, secrete cytokines, and interact with tumor cells.

Blocking the PD-1/PD-L1 signaling by using immunotherapy drugs can prevent tumor immune escape, increasing the anti-tumor activity of immune cells by improving T-cell activation and cytotoxic T cell killing activity [108,123,126].

Even if immunotherapy is a promising option for advanced and castration-resistant tumors, not all the patients respond. Immunotherapy-refractory cases can be due to “adaptive” or “innate” immune resistance [178]. The “adaptive immune resistance” represents a situation in which PD-L1 upregulation is driven by proinflammatory molecules produced by immune cells. IFN-γ and other soluble factors of the tumor microenvironment were able to upregulate PD-L1 in PC cells [10,13,94,103,106,112,119,126,128,139,152]. Conversely, when the “innate immune resistance” occurs, tumor cells autonomously upregulate PD-L1, under the influence of aberrant oncogenic pathways and without the induction of microenvironmental factors. In PCs, androgen ablation and loss of *PTEN* have been hypothesized as possible mechanisms of “innate immune resistance”, but data were discordant [94,179,180]. Different strategies have been tested to overcome the resistance of PC cells to immunotherapy, such as the combination of multiple immune checkpoint inhibitors, or the association with chimeric antigen receptor T (CAR-T) cells therapy, oncolytic adenoviral vectors expressing immunomodulatory molecules, Stimulator of Interferon Genes (STING) agonists, TLR9 agonists, or epigenetic drugs (such as histone deacetylase inhibitors) [181,182]. Further information about experimental treatments in pre-clinical models and other promising therapy approaches are better described in other parts of our systematic literature review (see Materials and Methods).

### 3.2. Intratumoral Lymphocytes

The most characterized molecular interaction is between PD-L1 expressed on antigen-presenting cells and PD-1 expressed on T-cells: after PD-1/PD-L1 binding, the “Src homology region 2-containing protein tyrosine phosphatase” SHP-1 and SHP-2 are recruited on the PD-1 intracellular tail at its immunoreceptor tyrosine-based inhibitory and switch motifs. Due to the spatial proximity of the PD-1 tail with the T-cell receptor (TCR) complex, these phosphatases block the activation of signaling downstream to TCR by interacting with ZAP70, PI3K-AKT and RAS pathways. SHP2 activates the signal transduction (including the JAK/STAT pathway) of various growth factors and cytokines: it is an oncoprotein promoting proliferation and survival, but it may also act as a tumor suppressor in some tumors [143,183]. In PC cell lines, high SHP2/STAT3 and low SHP1/STAT1 (phosphorylated or not) expression were reported. At least in some PC cell lines, SHP2 phosphorylated STAT1 (negatively regulating HLA-ABC and PD-L1 expression) and ERK (activating this pathway). SHP2 depletion was associated with increased T-cell activation by co-culture of allogeneic healthy donor peripheral blood monocytes with SHP2 siRNA-pretreated tumor cells [143].

Despite the cytoplasmatic protein tail lacks the canonical motifs for signal transduction, PD-L1 reverse intracellular signaling has been recently demonstrated in immune cells, lymphomas and solid tumors. Some non-canonical signaling transduction motifs have been identified in the intracellular tail, being involved in cancer cell proliferation and survival, inhibition of autophagy, mTOR activation, and IFN-β toxicity [158,184].

PC frequently shows scant lymphocytic infiltrates or T-cell exclusion, while T-cells are restricted to the adjacent stroma and benign areas without direct contact between effectors and cancer cells. Moreover, impaired antigen presentation in tumors leads to inadequate activation and boosting of T cells [79]. Indeed, tumor cells can express molecules (such as IDO and PD-L1), impairing the CD8+ cytotoxic T cells activity [79]. Upregulation of PD-L1 on tumor and immunosuppressive cells can also represent a result of a productive antitumor immune response [147,148]. Tumor-specific CD8+ T cells may be present but exhausted, as demonstrated by expression of PD-1 and T-cell immunoglobulin and mucin-domain containing-3 (Tim-3) [79]. Furthermore, the identifiable immune cells usually have anergic and immunosuppressive phenotypes, including Tregs, M2-polarized TAMs, and MDSCs. Regulatory T cells include CD4+/CD25+/FoxP3+ Tregs and a subset of CD8+/CD122+ T cells which are critical in maintaining the peripheral self-tolerance (avoiding autoimmunity) by suppression of CD8+ cytotoxic T cells and IFN-γ secretion; however, they also favor tumor immune-escape and progression, leading to poor outcomes: they are increased in peripheral blood and microenvironments of various tumors.

In some studies, PCs with poor prognosis had low infiltration of T- and dendritic cells, as well as high Tregs and TAMs levels; conversely, increased intratumor NK-cell infiltrates are associated with a low risk of progression. Improper TILs functionality (anergy, exhaustion or senescence) may also influence PC-immunosuppression. Activated NK cells release secretory lysosomes containing cytotoxic proteins (granzymes, perforin, etc.) to kill tumor cells, while cytotoxic T lymphocytes kill target cells by releasing granzymes or inducing Fas ligand-mediated apoptosis [108].

PD-L1 and VISTA are inhibitory molecules that can suppress murine and human T-cell responses. VISTA may represent a compensatory inhibitory pathway in PC after ipilimumab therapy [80]. Indeed, Gao et al. found significantly greater protein expression of PD-1, PD-L1, and VISTA in PC tissues treated with ipilimumab. PD-L1 and VISTA were upregulated in subsets of post-treatment intratumoral macrophages (CD163 + and ARG1 +, suggesting a M2-like phenotype) and in blood monocytes. High PD-L1 expression was found in CD4+ T cells, CD8+ T cells, and CD68+ macrophages [81], while VISTA may also be expressed by CD8+ cells [30].

### 3.3. NK Cells

Rare studies investigated the potential correlations between the NK cell-mediated cytotoxicity (NKCC) and PD-L1 expression in PC. Highly effective NK cells may be associated with good prognosis in metastatic CRPC-patients [185,186]. NKCC is independent of the tumor mutation burden, and NK cells play a pivotal role in exerting antigen-independent, innate immune responses, representing the early defense against cancer [135,185,186,187].

In addition to PD-L1 upregulation, suppression of NK group 2D (NKG2D)-activating ligands (including ULBP1, ULBP2, ULBP3, MHC class I chain-related molecules A and B, MICA and MICB) may represent another way of tumor escape from NKCC [188]. Conversely, upregulation of NKG2D ligands promotes the anti-tumor activity of NK cells [189]. Hypoxia inhibited the expression of NKG2D ligands on the surface of the PC cell lines, and thus the killing of tumor cells by activated NK cells [65]. IL-6 upregulates PD-L1 and simultaneously downregulates NKG2D ligands [135]. Treatment of CRPC cells and NK cells with THP-1 (conditioned media) revealed the same effect, decreasing the susceptibility of tumor cells to the NKCC [134,135].

Unlike T cells, NK cells did not express high PD-1 levels; however, PD-1 exxpression can be increased upon incubation with tumor cells [134,135]. Leptin is a protein hormone expressed by adipocytes, with a role in regulating body weight, metabolism, and reproductive function: using conditioned media in vitro experiments on PC cells, some authors suggested that, like IL-6, this adipokine can induce resistance of CRPCs to NK cell action by activating the JAK/STAT3 pathway and regulating the PD-L1NKG2D ligand levels. Experimental studies suggested that inhibition of leptin or IL-6-JAK/STAT3 signaling in CRPC-cells enhances the anti-tumor NKCC via alteration of PD-L1/NKG2D-ligands levels [65,134,136].

### 3.4. Tumor-Associated Myeloid Cells

Tumor-infiltrating myeloid cells such as TAMs or MDSCs secrete anti-inflammatory cytokines and are important components of the immunosuppressive TME, representing potential sensitive therapeutic targets for immunomodulation. Stress and inflammation can trigger and/or sustain STAT3 activity in PC cells and, especially, in tumor-associated myeloid cells [97]. Myeloid cells can be found in either low- or high-grade PCs, potentially having an early and sustained role in tumor progression. Myeloid cell infiltration into malignant tissues results from chemokines-mediated attraction, that is also regulated by tumor-stroma interaction [97,184].

TAMs can contribute to tumor progression, promoting genetic instability, supporting metastasis, and sometimes antagonizing the response to chemo- or radio-therapy. In PCs with numerous PD-L1+ cells, Scimeca et al. found a reduction and M2 polarization of TAMs, linked to the expression of PD-L1 [48]. M2 TAMs have an anti-inflammatory protumor effect, positively associated with aggressive pathologic features, malignant progression and recurrence after prostatectomy [47]. Indeed, they may exert immunosuppressive effects on T-cells through cytokine production and the PD-1/PD-L1 pathway, as by recruiting other immunosuppressive cells (MDSCs, immature dendritic cells, Tregs). Some authors found that TAMs express PD-L1/2, while others reported no change in the expression of immune checkpoint regulators (PD-L1 and B7-H3) in HLA-DR-/CD15+ MDSCs derived from patients’ blood [45]. TAM may also decrease the NKCC to CRPC cells; NKG2D receptor on NK cells can be modulated by immunosuppressive TAMs, which may induce tumor immune escape from NK cell action [48,135]. FGF favors tumor cell dissemination, angiogenesis, and metastases in various types of solid cancers: elevated FGF levels correlated with an increase of M2 TAMs [45].

An experimental study found that EZH2 (Enhancer Of Zeste 2 Polycomb Repressive Complex 2 Subunit) inhibitors did not dramatically alter MDSC tumor infiltrate populations, but significantly reprogrammed TAM infiltrates, decreasing tumor-promoting M2 TAMs and increasing tumor-inhibiting M1 TAMs [155]. Colony-stimulating factor (CSF)-1 controls the differentiation, proliferation, and survival of macrophages by binding to CSF1 receptor, expressed on macrophages: increased Δ133TP53β mRNA in PC was characterized by an immunosuppressive phenotype and increased frequency of PD-1, PD-L1, and CSF1 receptor-positive cells [40].

Cell death releases Toll-like receptor 9 (TLR9) ligands (mitochondrial DNA) while the TLR9/NF-kB–induced secretion of IL6 activates the STAT3 pathway. High TLR9 expression and STAT3 activation in immunosuppressive polymorphonuclear MDSCs (PMN-MDSC) accumulate in the blood of progressing metastatic/CRPC-patients. Moreover, in PC models, STAT3 activity in tumor-infiltrating MDSCs correlated with increased PD-L1 levels and elevated plasma levels of IL6-type cytokines (such as LIF), suggesting a potential cross-talk mechanism promoting tumor immune evasion [190]. The TLR9+ PMN-MDSCs block T-cell proliferation and activity [130]. *MYC* oncogene expression and/or combined deletion of *PTEN/SMAD4* or *PTEN/TP53* also cause expansion of TAMs and MDSCs, promoting tumor immunotolerance and vascularization [130]. MDSC-secreted IL-23 is a potential driver of CRPC (at least in a subset of patients) via activation of AR signaling [191].

The abundance of circulating MDSCs correlated with prostate-specific antigen levels and metastasis in PC patients [81].

In the study of Sharma et al. [88], CD14- granulocytic MDSCs were significantly more common (*p* < 0.01) in pelvic lymph nodes than peripheral blood: this subgroup exhibited a high degree of immunosuppressive activity, as to the high STAT3 levels and PD-L1/2 expression. Conversely, no significant accumulation of CD4+/FOXP3+ Tregs was reported in pelvic lymph nodes, and CD14+ monocytic MDSCs were identified at rates similar to those detected in the peripheral blood of PC-patients.

PTX3 (TSG-14) (member of the long-pentraxin subfamily) is released by peripheral blood leukocytes and myeloid dendritic cells in response to proinflammatory stimuli (such as TNF-α and IL-1β), but it may also have a role in inflammatory-related carcinogenesis, being expressed by either PC cells or intratumoral inflammatory infiltrates, especially macrophages. PTX3 overexpression in PC and other tumor types was considered as an unfavorable prognostic factor. Scimeca et al. reported increased numbers of PD-L1+ and PTX3+ cells in PCs (compared to benign lesions): PTX3 expression showed an inverse correlation with the number of PD-L1+ PC-cells. PD-L1+ PCs revealed decreased PD1+ lymphocytes and M2 TAMs [48].

### 3.5. Dendritic Cells (DC) and Stromal Cells

Tumor-associated stroma may be an immunosuppressive barrier to anticancer immunity, negatively influencing PC-progression by eliciting myeloid cell migration and altering their differentiation into fully functional DCs.

In the study of Spary et al., stromal cells produced significantly higher levels of CCL2, IL-6 and TGF-β than epithelial cells. Indeed, PC-cells have minimal chemoattraction for myeloid cells; conversely, PC-stromal cells attract monocytes via CCL2 secretion in early PCs. Intratumoral CD68+ myeloid cells correlated with increased risk of PC-recurrence. CCL2 can be produced by various cell types, including epithelial, fibroblastic, endothelial and smooth muscle cells. CCL2 also activates CD11b+ monocytes, enhancing their IL-6 expression. IL-6 favors PD-L1 upregulation, and reciprocally increases CCL2 tissue levels, resulting in an amplification loop that promotes monocyte infiltration, malignant cell proliferation and tumor survival [97].

Fibroblast-derived IL-6 has been shown to affect the differentiation of monocytes into macrophages. Moreover, stromal-derived factors also promote differentiation of monocytes to DCs, inducing an immunosuppressive phenotype in DCs (CD14+/CD16+/CD68+/CD124+/CD209+; PD-L1 overexpression). Thus, DCs become incapable of cross-presenting tumor antigens to T cells, inhibiting T-cell responses [97].

Stromal conditioned media may also influence the cytokine release from DCs. In IL-4 and GM-CSF treated monocytes, the immune-regulatory effect of stromal cells is mediated via the STAT3 pathway. In PCs, IL-6 can be expressed by stromal and tumor cells, influencing the DC differentiation and function, which are inhibited by STAT3 activation. IL-10 generated CD14+ DC in vitro, favoring high PD-L1 expression levels. Monocytes exposed to stromal factors did not produce detectable amounts of IL-10; however, upon lipopolysaccharide stimulation, stromal factor-generated DCs (sDC) induce significantly more IL-10 and less IL-12 than their conventional DC-counterparts. sDC failed the cross-presentation of tumor-antigens to CD8+ T cells and suppressed the T-cell proliferation. sDCs showed significantly increased PD-L1 levels in a primarily STAT3 and IL-6-dependent manner. Inhibition of STAT3 restored CD14 downregulation during DC differentiation in the presence of PC-stromal cell-conditioned medium, and significantly inhibited the upregulation of PD-L1 on DCs [97].

### 3.6. Adipocytes

Obesity increases the risk of recurrence and castration-resistance in PC: periprostatic adipocytes may have paracrine effects on PC-tumor cells progression, by secretion of hormones and adipocyte-derived chemokines (adipokines) [136,192,193,194]. The obese state may favor a chronic inflammatory condition: adipokines are involved in inflammatory modulation (also attracting macrophages and T-cells), glucose and lipid metabolism, hypertension, or insulin sensitivity, and may also have a role in cancer progression.

In vitro experiments on PC cells suggested that adipokines/proinflammatory cytokines such as leptin (a protein hormone expressed by adipocytes, with a role in regulating body weight, metabolism, and reproductive function) and IL-6 can induce resistance of CRPCs to NK cell action via the JAK/STAT3 pathway, thus regulating the PD- L1/NKG2D ligand levels. IL-6 may also be involved in obesity-related insulin resistance: plasma IL-6 levels positively correlated to weight gain in humans [136].

### 3.7. Comments on the Intracellular Signaling Pathways Involved in PD-L1 Expression

In the perspectives of this review, experimental studies performed on human and mouse PC-cell lines revealed that the intracellular JAK/STAT, ERK/MEK, Akt-mTOR, NF-kB, and WNT pathways were involved in PD-L1 regulation in PC. Activation of ERK, NF-kB and JAK/STAT pathways leads to PD-L1 expression, regardless of the tumor sample (primary vs. metastatic) and of the androgen responsiveness/resistance of the analyzed PC-cell lines.

DNA double-strand breaks activate the STAT signaling [195,196]. STAT3 plays an important role in wound healing and tissue repair and it is frequently overactivated in malignant tumors, resulting in inflammation-driven repair, promotion of drug resistance and tumor progression. In some tumors (such as melanoma), inactivating mutations of JAK/STAT signaling are responsible of immunotherapy resistance [197,198,199], while data in PC are discordant. Persistent STAT activation was found in the majority of human PC-cell lines used for in vitro experiments, especially in metastatic androgen-resistant PC cell lines (such as PC3, DU145 and Vcap) [200]. A hyper-phosphorylation of STAT was also observed in human PC samples: STAT activation was evident in tumor areas, lacking in the normal tissue [200]. Constitutive STAT3 activity can result in tumor progression toward the CRPC phenotype and may be associated with poor overall survival [130]. An inverse correlation between activation of JAK/STAT pathway and AR expression was described in PC cell lines [200]. STAT3 levels may be also elevated in CRPC patients’ serum, leading to transcriptional activation of AR [115,201,202]. Combined inhibition of STAT3-PD-L1 signaling can suppress CRPC immune escape, enhancing the NKCC [65].

Various cytokines (such as IL-6, IFN-α, β, and γ) stimulate the JAK/STAT pathway, regulating cell proliferation, migration, differentiation, and inflammation. The JAK/STAT3 signaling is frequently over-activated in PC-cells, upregulating PD-L1 expression, suppressing the antigen presentation of DCs, and mediating other immunosuppressive effects. The binding of IL-6 to its receptor (IL-6R) activates the JAK/STAT signaling with STAT3 phosphorylation (pSTAT3): PD-L1 overexpression is significantly correlated to the pSTAT3 status [115]. In some studies, pSTAT3 and IL-6R expression were detected in 95% of metastatic CRPCs, being higher in bone vs. lymph node/visceral metastases [203]. Moreover, the most undifferentiated stem-like PC cells expressed the highest levels of IL-6 and IL-6R: blockade of JAK/STAT3 signaling could inhibit colony-forming and tumor initiation [204]. In addition, IL-6/JAK/STAT3 signaling could induce “pituitary tumor transforming gene 1” (PTTG1) overexpression with subsequent induction of epithelial-mesenchymal transition, increasing the cancer stem cell population in LNCaP androgen-dependent PC cell lines. Moreover, STAT3 inhibition significantly increased the sensitivity of the DU-145 androgen-independent CRPC-cell line to bicalutamide (antiandrogen therapy) [126,203,204,205,206].

Ihle et al. reported that immune cells of blastic bone metastasis were enriched for pSTAT3, and multiple checkpoint inhibitor targets (B7-H4 VTCN1, PD-L1, PD-1, VISTA, OX40L, IDO-1 and ICOS CD278) [31]. Conversely, in lytic-type lesions, immune cells were enriched for phosphorylated AKT activity and components of the PI3K-AKT pathway.

The JAK/STAT signaling seems involved in PD-L1 expression not only in acinar PCs, but also in small cell neuroendocrine PCs through various mechanisms, including epigenetic methylation [111]. Owen et al. documented a loss of type I INF signaling in proliferating PC cells of bone metastases: this loss was associated with the suppression of tumor immunogenicity. The authors demonstrated that the restoration of tumor INF signaling by histone deacetylase inhibition increased the tumor cell visibility, promoted long-term antitumor immunity, and blocked cancer growth in a syngeneic mouse model [182].

Mitogen-activated protein kinases (MAPKs) are serine/threonine kinases mediating intracellular signaling associated with a variety of cellular activities including cell proliferation, differentiation, survival, death, and transformation. JNK and ERK are two of the major signal transducers involved in this pathway: JNK is generally associated with apoptosis induction, while ERK1/2 are frequently correlated to mitogenesis and inversely related to apoptosis [207]. JNK activity is altered in PCs, while ERK1/2 is typically phosphorylated in metastatic CRPCs [207,208,209,210]. The main oncogenes involved in this pathway rarely harbor genetic mutations, while amplifications of MAPK genes were found in about 1/3 of cases [211,212]. RAS/MAPK pathway activating mutations are probably a “second hit” to the PTEN/PI3K/AKT pathway loss in metastatic PC [211].

*PIK3CA* mutations induce AKT–mTOR activation, increasing PD-L1 expression [156]. *PTEN* upregulation inhibits mTOR, and PD-L1 has been documented in mice injected with PC-cells overexpressing chemerin (a *PTEN*-activator). The AKT-mTOR inhibition and chemerin-induced PD-L1 downregulation significantly reduced the tumor growth [105].

NF-kB factors are regulators involved in inflammatory and immune response, apoptosis and cell growth. NF-kB activation has been observed in various tumors, including PC [213,214,215]. NF-κB-inhibiting molecules can inhibit the growth of the androgen-independent PC cell lines; combination treatments with NF-κB and AR inhibitors have shown promising results [216,217]. Moreover, several protein kinase C proteins have been implicated in PC development, and considered as putative therapeutic targets [216].

Finally, the Wnt signaling is involved in the embryological development and maintenance of stem cell populations. When the Wnt ligand (a secreted glycoprotein) binds to Frizzled receptors, a large cell surface “Wnt receptor complex” (WRC) is formed with LRP5/6. After β-catenin (hallmark of Wnt signaling) is stabilized by the activated WRC, it is translocated into the nucleus, where it binds to LEF/TCF transcription factors, displacing co-repressors and recruiting co-activators to Wnt target genes (including oncogenes such as *C-MYC* or *CCND*1). The Wnt/β-catenin pathway could regulate PD-L1 expression in a direct manner, or indirectly via c-MYC binding to the PD-L1 promoter (activating its expression). CTLA-4 is also a direct target gene of Wnt/β-Catenin in melanoma: it increases the production of IFN-γ by CD8+ and CD4+ TILs [218,219].

Epigenetic and genetic alterations in the Wnt pathway have been observed in several tumors, including PCs [220]. However, activating *CTNNB1* (β-Catenin) mutations have been rarely detected in PCs [221]. Loss of heterozygosity and mutations of *APC* gene may also influence this pathway [222]. Activating alterations have been observed more frequently in CRPCs than in treatment-naïve tumors [223,224,225]: targeting the Wnt/β-catenin signaling may represent a potential therapeutic strategy against CRPCs.

PC can be associated with abnormal cholesterol metabolism and hypercholesterolemia: the low-density lipoprotein (LDL) receptor-related protein (LRP) family regulates lipid metabolism by receptor-mediated lipoprotein endocytosis. LRP1 and LRP5 could promote PC-progression. LRP11 over-expression in PC-cell lines activates β-catenin signaling, inducing PD-L1 expression: these effects seemed unrelated to the AR status. In addition, LRP11 induced immunosuppression in a co-culture system [117].

CTHRC1 has been proposed as a pivotal tumor promoter and activator of the planar cell polarity pathway via stabilization of the Wnt-receptor complex. It seems upregulated in various cancers (pancreas, stomach, liver, esophagus). In a PC series, CTHRC1 overexpression was associated with worse disease-free-survival, upregulation of PD-1 and PD-L1, increased inflammatory infiltrates (B cells, CD4+ T cells, macrophages, neutrophils and dendritic cells) and increased expression of matrix metalloproteinase-9, mucin 1 and *SLCO2B1* (solute carrier organic anion transporter family member 2B1) genes [120].

## 4. Materials and Methods

In health care, increasing attention has been paid to systematic literature reviews (SLRs) and meta-analyses, which are used by clinicians to keep themselves up-to-date. Moreover, SLRs are often a starting point for further trials or for developing clinical guidelines. Finally, granting agencies may base the justification for research financial support on SLRs. For these reasons, impacting health care journals frequently ask contributing authors to conduct their SLRs according to the PRISMA guidelines (http://www.prisma-statement.org/; accessed on 8 May 2021), which include an evidence-based minimum set of items for reporting and represent a useful aid for a critical evaluation of the submitted manuscripts. So, we have conducted our SLR according to these guidelines, searching in multiple databases, as previously described in the various topics/contexts in which these guidelines are applicable [226,227,228,229,230,231,232,233,234,235,236,237,238,239,240,241,242,243,244,245,246,247,248,249,250,251,252,253,254,255,256,257,258].

Our study aimed to answer the following PICOS (Population, Intervention, Comparison, Outcomes) questions:

- Population: patients, tumor cell lines, or mouse models included in studies concerning the role of PD-L1 in PC;
- Intervention: any type of treatment;
- Comparison: no comparisons are expected;
- Outcomes: patient’s status at last follow-up (no evidence of disease, alive with disease, dead of disease), response to therapy, biochemical recurrence-free survival, metastasis-free survival, cancer-specific survival, disease-free survival, clinical failure-free survival, overall-survival, progression-free survival; for experiments on PC cell lines and mouse models, any reported effect on cancer and immune cell migration, proliferation, viability, growth, resistance/response to therapy, cytotoxic/anti-tumor activity, PD-L1 expression, and mice/cell lines survival.

Study design: retrospective observational study (case series/reports, clinical trials, experimental studies).

Eligibility/inclusion criteria: experimental studies (tumor cell lines, mouse models) or clinic-pathologic studies on human patients concerning the role PD-L1 in PCs.

Exclusion criteria: tumors not arising from the prostate; non-carcinomatous histotypes; studies not examining PD-L1; cases with uncertain diagnosis; review articles without new cases.

Information sources and search strategy: we searched for (PD-L1 AND (prostate OR prostatic) AND (adenocarcinoma OR adenocarcinomas OR cancer)) in Pubmed (all fields), Web of Science (Topic/Title), and Scopus (Title/Abstract/Keywords) databases. No limitations or additional filters were set. The bibliographic research ended on 8 May 2021.

Study selection: two independent reviewers selected the studies using a 2-steps screening method. In the 1st step, screening of titles and abstracts was performed to verify the eligibility/inclusion criteria, excluding irrelevant studies. In the 2nd step, full texts of relevant articles were screened by the 2 reviewers to verify study eligibility and inclusion criteria, avoiding duplications. Two other authors performed a manual screening of reference lists, to search for additional relevant papers. Finally, two authors checked the extracted data.

Object of the systematic review: (1) to update and summarize the literature concerning the role of PD-L1 on PC cells; (2) to report any information about clinic-pathological features, treatment strategies, and patients’ outcomes.

Data collection process/data items: study-related (authors and year of publication) and case-related (tumor stage at presentation, Grade Group, type of specimen, treatment, test methods and results of PD-L1 expression, follow-up and outcomes, experiment type).

Statistical analysis: the collected data were reported as continuous or categorical variables. Categorical variables were summarized by frequency and percentage, while continuous variables by ranges, mean and median values. Time-to-recurrence was the time from primary treatment to disease recurrence. The survival status was the time from primary treatment to the last follow-up.

To better present the results of our systematic literature review and discuss the multiple interesting facets of PD-L1 expression by PC in detail, we have divided the presentation and discussion of our results into different articles, representing independent chapters of our work. They highlight various subtopics, including: PD-L1 immunohistochemical expression in PC cases, with discussion of pre-analytical and interpretation variables; correlations of PD-L1 expression with clinic-pathological features in PC patients; genetic and epigenetic regulation of PD-L1; data of pre-clinical studies (cell lines, mouse models) about the effects of experimental treatments on PD-L1 expression in PC cells; investigated correlations of PD-L1 expression with the status of mismatch repair system, *BRCA*, *PTEN* and other main genes in PC; PD-L1 expression in liquid biopsy samples; information of clinical trials, etc. We address the Readers to the other papers for further details on these subtopics [259,260,261,262,263,264,265,266,267,268,269,270,271,272,273,274,275,276].

## 5. Conclusions

TME includes immune (T, B, NK, dendritic), stromal/mesenchymal, endothelial, adipocytic cells, extracellular matrix, and cytokines/chemokines/soluble factors, which regulate various intracellular signaling pathways in tumor cells. TME influences PC growth and progression, enhancing tumor cell immune-evasion also through the activation of the PD-1/PD-L1 pathway. Immune-escape mechanisms of PC include suppression/exhaustion of tumor infiltrating cytotoxic T CD8+ lymphocytes, inhibition of tumor suppressive NK cells, increase in immune-suppressive immune cells (M2 macrophages, MDSCs, dendritic, stromal, adipocytic cells). IFN-γ, TGF-β, TNF-α, IL-6, IL-17, IL-15, IL-27, complement factor C5a, and other soluble molecules secreted by TME components, as well as and hypoxia, influence PD-L1 regulation. Experimental studies using human and mouse PC cell lines (derived from either androgen-sensitive or androgen-resistant tumors) revealed that the JAK/STAT, ERK/MEK, Akt-mTOR, NF-kB, and WNT intracellular pathways were involved in PD-L1 upregulation in PC. Immunotherapy drugs can increase the anti-tumor activity of immune cells, preventing tumor immune-escape.

## Figures and Tables

**Table 1:** Extracellular factors involved in the regulation of PD-L1.

Factor	Experiment Type	Cell Lines	Effects on PD-L1	Studied Effect
**Check point**				
Soluble PD-1 [149]	Co-culture and Docetaxel treatment	DU145 and Jurkat	Act.	↑ Docetaxel resistance
**Soluble factors produced by stromal cells**				
AREG [121]	Conditioned medium	DU145, PC3, LNCaP	↑	↑ Proliferation, migration and invasion
IL-6 [97]	Conditioned medium	Dentritic cells	↑	//
**Soluble factors produced by adipocytes**				
Not identified [136]	Co-culture with conditioned medium	C4-2 and NK; CWR22Rv1 and NK	↑	↓ NK cytotoxicity
**Soluble factors produced by macrophages**				
Not identified [134]	Co-culture with conditioned medium	C4-2 and NK; CWR22Rv1 and NK	↑	↓ NK cytotoxicity
**Cytokine/Chemokine and** **Complement factors**				
IL-27 [151]	Treatment	PC3	↑	//
IFN-γ [10,13,94,103,112,119,128,139,152]	Treatment	PC3	↑	//
IFN-γ [94]	Treatment	Vcap CWR22Rv1, E006AA	↑	//
IFN-γ [126]	Treatment	TRAMP-C2	↑	//
IFN-γ [13]	Treatment	LASCPC, NCI-H660	↑	//
IFN-γ [126]	Treatment	DU145	=	//
IFN-γ [13,94,128]	Treatment	LNCaP	=	//
IFN-γ [94]	Treatment	LAPC-4	=	//
IFN-γ [13]	Treatment	BPH1, C4-2, CWRR-1	=	//
IFN-γ [10,94,112,119,152]	Treatment	DU145	↑	//
IFN-γ [112]	Treatment	TRAMP-C2 Ras	↑	//
IFN-γ [106]	Treatment	TRAMP-C1, MyC-CaP	↑	//
Ab anti-IL-6 [134,135,136]	Treatment	C4-2, CWR22Rv1	↓	//
IL-6 [135]	Treatment	C4-2, CWR22Rv1	↑	//
IL-17 [145]	Treatment	LNCaP	↑	//
TNF-α [145]	Treatment	LNCaP	↑	//
Chemerin [105]	Treatment of co-culture	DU145 and T	↓	↑ T cytotoxicity
C5a [18]	Treatment	PC3, C4-2	↑	//
**Hypoxia**				
[65]	Co-culture	C4-2 and NK; CWR22Rv1 and NK	↑	↓ NK cytotoxicity

Act. Activation; **↑** Upregulation; **↓** Downregulation; = No alteration; // No effect was investigated.

**Table 2:** Effects of IFN-γ stimulation on PD-L1 in PC-cell lines.

Cell Lines	Origin	IFN-γ Dose	Treatment Time	Detection Method	Effect on PD-L1
BPH1 [13]	Human	50 mg/mL	24 h	WB	=
CWR22Rv1 [94]	Human	100 U/mL	48 h	FC	↑
CWRR-1 [13]	Human	50 mg/mL	24 h	WB	=
C4-2 [13]	Human	50 mg/mL	24 h	WB	=
DU145 [126]	Human	0.5–10–20 ng/mL	48 h	FC	=
DU145 [10]	Human	Not reported	Not indicated	RT-PCR, FC	↑
DU145 [94]	Human	100 U/mL	48 h	FC	↑
DU145 [112]	Human	10 ng/mL	24 h	WB, FC	↑
DU145 [119]	Human	100 U/mL	48 h	FC	↑
DU145 [152]	Human	10 ng	24 h	FC	↑
E006AA [94]	Human	100 U/mL	48 h	FC	↑
LASCPC [13]	Human	50 mg/mL	24 h	WB	↑
LAPC-4 [94]	Human	100 U/mL	48 h	FC	=
LNCaP [13]	Human	50 mg/mL	24 h	WB	=
LNCaP [94]	Human	100 U/mL	48 h	FC	=
LNCaP [128]	Human	10–100 ng/mL	24 h	FC	=
MyC-CaP [106]	Mouse	0.1–1–10 ng/mL	72 h	FC	↑
NCI-H660 [13]	Human	50 mg/mL	24 h	WB	↑
PC3 [10]	Human	Not reported	Not indicated	RT-PCR, FC	↑
PC3 [13]	Human	50 mg/mL	24 h	WB	↑
PC3 [94]	Human	100 U/mL	48 h	FC	↑
PC3 [103]	Human	20 ng/mL	24 h	WB, FC	↑
PC3 [112]	Human	10 ng/mL	24 h	WB, FC	↑
PC3 [119]	Human	100 U/mL	48 h	FC	↑
PC3 [128]	Human	10–100 ng/mL	24 h	FC	↑
PC3 [139]	Human	100 ng/mL	24 h	RT-PCR, WB	↑
PC3 [152]	Human	10 ng	24 h	FC	↑
TRAMP-C1 [106]	Mouse	0.1–1–10 ng/mL	72 h	FC	↑
TRAMP-C2 [126]	Mouse	0.5–10–20 ng/mL	48 h	FC	↑
TRAMP-C2 Ras [112]	Mouse	10 ng/mL	24 h	WB, FC	↑
Vcap [94]	Human	100 U/mL	48 h	FC	↑

**↑** Upregulation/increase; = No alteration; FC: flow cytometry; RT-PCR: Real-Time Polymerase Chain Reaction analysis; WB: Western blot analysis.
